# Application of 3D printing and distributed manufacturing during the first-wave of COVID-19 pandemic. Our experience at a third-level university hospital

**DOI:** 10.1186/s41205-021-00097-6

**Published:** 2021-03-08

**Authors:** Rubén Perez-Mañanes, Sonia García-de San José, Manuel Desco-Menéndez, Ignacio Sánchez-Arcilla, Esmeralda González-Fernández, Javier Vaquero-Martín, Javier Pascau González-Garzón, Lydia Mediavilla-Santos, Diego Trapero-Moreno, José Antonio Calvo-Haro

**Affiliations:** 1 Advanced Planning and 3D Manufacturing Unit, Hospital General Universitario Gregorio Marañón, Madrid, Spain; 2 Department of Orthopaedic Surgery and Traumatology, Hospital General Universitario Gregorio Marañón, Madrid, Spain; 3 Faculty of Medicine. Department of Surgery, Universidad Complutense, Madrid, Spain; 4 Instituto de Investigación Sanitaria Gregorio Marañón, Madrid, Spain; 5 Deputy Hospital Management, Hospital General Universitario Gregorio Marañón, Madrid, Spain; 6 Department of Bioengineering and Aerospace Engineering, Universidad Carlos III, Madrid, Spain; 7 Department of Labour Risks Prevention, Hospital General Universitario Gregorio Marañón, Madrid, Spain; 8 Supply Management, Hospital General Universitario Gregorio Marañón, Madrid, Spain

**Keywords:** 3D printing, distributed manufacturing, POC manufacturing, COVID-19, personal protective equipment, face shields, ear savers, ventilatory support, swabs

## Abstract

**Background:**

3D printing and distributed manufacturing represent a paradigm shift in the health system that is becoming critical during the COVID-19 pandemic. University hospitals are also taking on the role of manufacturers of custom-made solutions thanks to 3D printing technology.

**Case Presentation:**

We present a monocentric observational case study regarding the distributed manufacturing of three groups of products during the period of the COVID-19 pandemic from 14 March to 10 May 2020: personal protective equipment, ventilatory support, and diagnostic and consumable products. Networking during this period has enabled the delivery of a total of 17,276 units of products manufactured using 3D printing technology. The most manufactured product was the face shields and ear savers, while the one that achieved the greatest clinical impact was the mechanical ventilation adapters and swabs. The products were manufactured by individuals in 57.3% of the cases, and our hospital acted as the main delivery node in a hub with 10 other hospitals. The main advantage of this production model is the fast response to stock needs, being able to adapt almost in real time.

**Conclusions:**

The role of 3D printing in the hospital environment allows the reconciliation of in-house and distributed manufacturing with traditional production, providing custom-made adaptation of the specifications, as well as maximum efficiency in the working and availability of resources, which is of special importance at critical times for health systems such as the current COVID-19 pandemic.

## Background

The exponential demand for technical and human resources caused by the COVID-19 pandemic has challenged public health, healthcare organization and hospital care, bringing health systems in many countries to the brink of collapse, with the added difficulty of coping with chronic shortages of personal protective equipment (PPE) and other essential medical supplies [1]. These problems, accentuated both by relocation and by the reduction in business activity due to a lack of supplies from abroad, have shown that it is necessary to have certain manufacturing capacities for the production and equitable supply of essential products, which are crucial for saving lives, as well as for curbing the socio-economic impact [2].3D printing is a technology that can lead this change in the manufacturing paradigm. It allows the design to be made anywhere in the world because the product as such moves in a digital format to any place, just by printing it with a 3D printer [3]. In this context of distributed manufacturing, the fact that products are obtained from the same machine at a fixed and predictable cost makes it possible to print at a location close to the end user, ensuring that they arrive in the shortest possible time [4]. The expiration of patents related to different 3D printing technologies such as FDM (Fused Deposition Modeling), the existence of new digital and social platforms, the possibility of collaborative projects ("crowdfunding"), and socio-cultural movements such as the Maker movement [5], have allowed people not only to consume, but also to make and share. Access to this technology during the pandemic has made it easier for professionals and patients, with the aim of seeking to improve people's health, to react to the shortage of equipment by promoting new production models that can quickly respond to a situation of complete uncertainty in health action with regard to such basic issues as transmission, lethality and ethical questions regarding the prioritisation of resources.

In these new production models, university hospitals can act as manufacturing centers using in-house 3D printing technology and distributed production, are part of hubs that allow them to reconcile in-house manufacturing and outsourcing services, strengthening collaborative work and ensuring that this type of manufacturing becomes the standard of care [6–9] and being able to efficiently adjust their resources, objectively identifying their limitations and creating external partnerships. New production models such as point-of-care manufacturing are contemplated, and this makes it possible to respond to needs for space or expensive facilities, to bring together the technical competence profile in industrial aspects, or to keep its portfolio of services up to date without depending on the obsolescence of machines and manufacturing materials, which is very rapid for this type of technology [6].

The aim of this case study is to identify the role of 3D printing technology in our university hospital during the first wave of the current COVID-19 pandemic.

## **Case presentation**

This is an observational, descriptive, monocentric case study that includes all the projects developed from the Advanced Planning and 3D Manufacturing Unit (UPAM3D) of the Hospital General Universitario Gregorio Marañón (Madrid, Spain) during the confinement period, as a consequence of the declaration of the state of alarm decreed in the country by the COVID-19 pandemic, between March 14 and May 10, 2020.

The following variables have been analyzed:

- Product type = Final product delivered.
- Number = Number of units of each of the final products delivered.
- Type of supplier = Manufacturers of the products.
- Type of delivery = Donation or purchases, referring to the delivery of the products made by the supplier, either donated or acquired by the hospital through a public purchase process.
- Date = Date of delivery of the final product to the place of destination.
- Destination= Place of delivery of the final product.

The variables have been described as number and percentage.

**Fig. 1:**
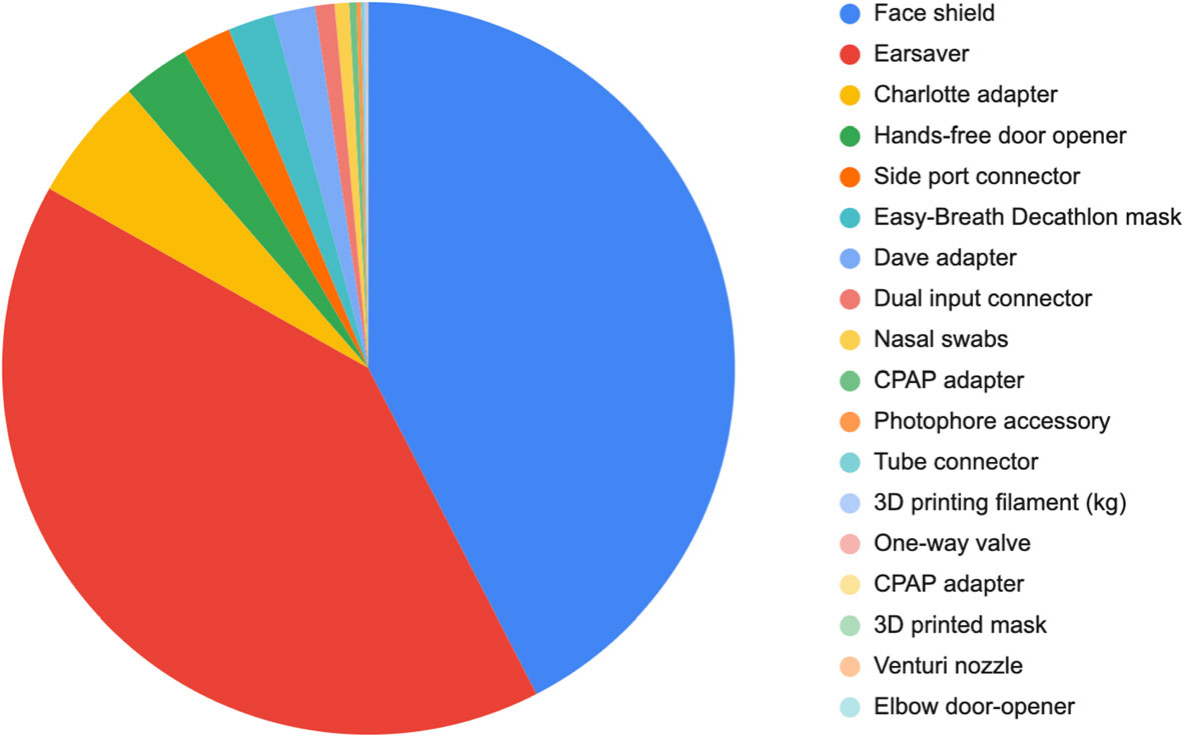
Products delivered

Networking during this period has enabled the delivery of a total of 17,276 units of products manufactured using 3D printing technology. (Fig. 1) All models manufactured had an end-use. Requirements were communicated to our laboratory on a daily basis and printed in-house or by external equipment according to availability and volume required. The models had a very intuitive use in most cases, or very technical use in some cases, such as adapters for ventilation systems.

**Fig. 2:**
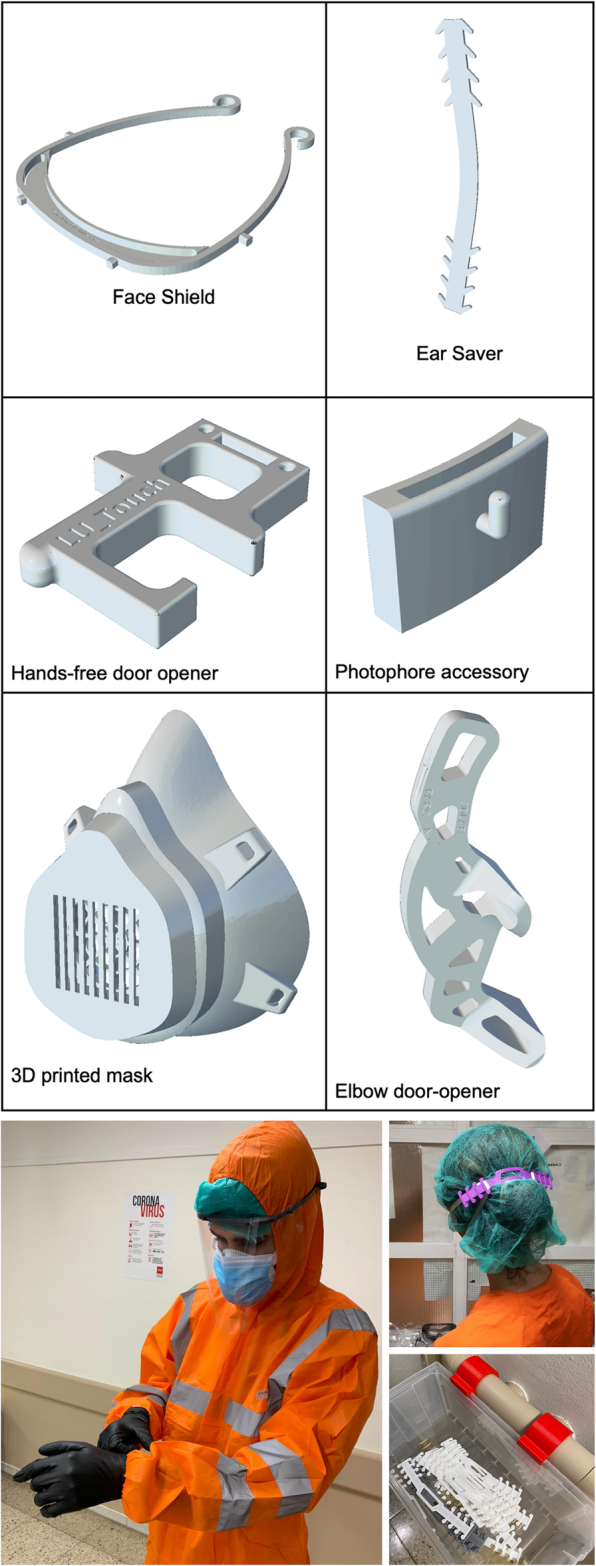
Images from the STL files and pictures of protection products

End products delivered can be divided into three main groups:

**1. Protection and prevention systems** (Fig. 2). This group includes the manufacture of face shields, 3D printed photophore accessories, ear savers, hands-free door openers, or prototypes of masks.

**Fig. 3:**
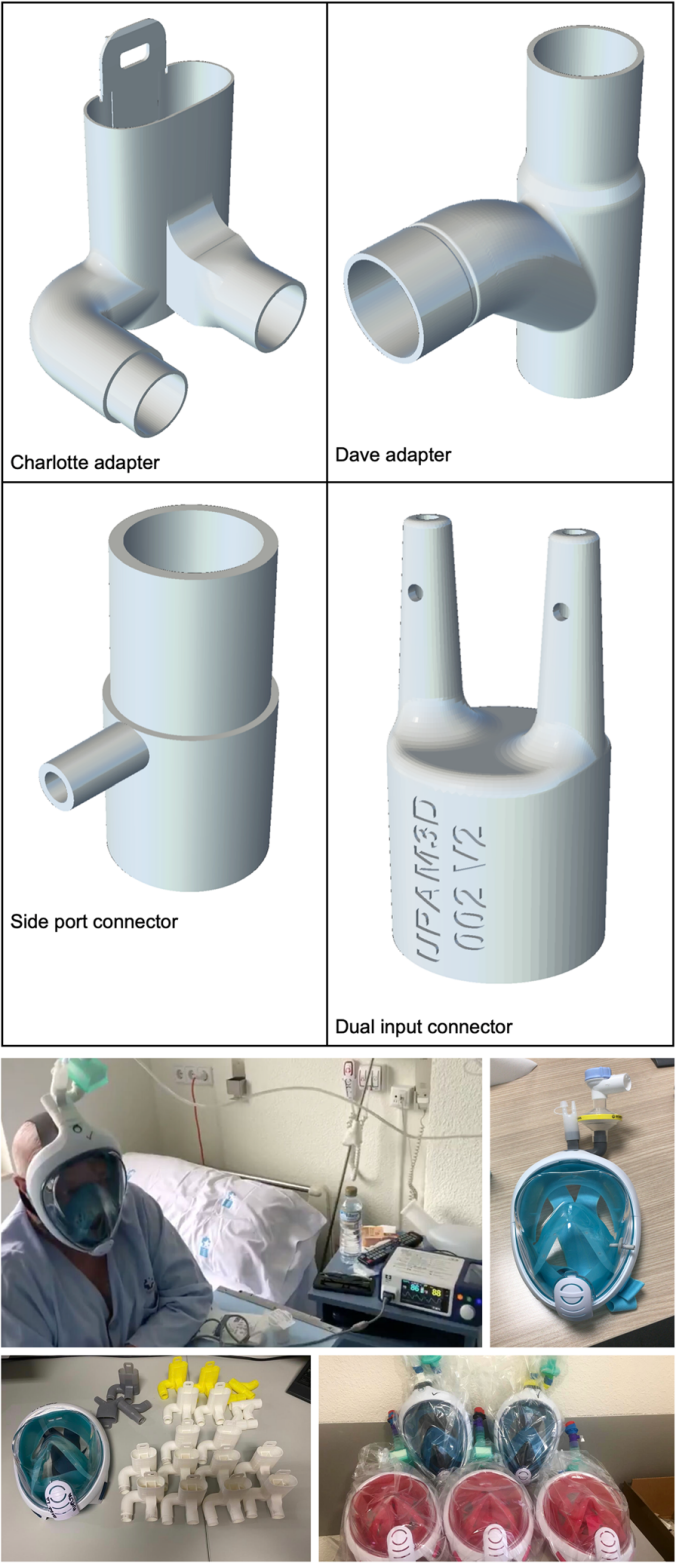
Images from the STL files and pictures of ventilation products

**2. Products for Ventilatory Support Therapies** (Fig. 3). The peak of hospital admissions due to the pandemic has caused a great demand for ventilation machines, with the consequent transitory lack, so we decided to modify non-sanitary material and sanitary material approved for other uses. This has allowed us to perform non-invasive treatment in those patients who needed respiratory support but not without sufficient stock of approved devices. In our center we have used the Easybreath® (Decathlon, France) diving mask system modified as a CPAP system (EASY-CPAP), having been necessary the acquisition of masks [10–13], the production of connector parts or supports, and the optimization of the design of other parts adding modifications to adapt it to the existing material in our center. Among the products manufactured, we have included connection parts for the masks and other parts for the ventilation systems such as straight connectors, connectors with side port for oxygen therapy or adapters for positive protection systems.

**Fig. 4:**
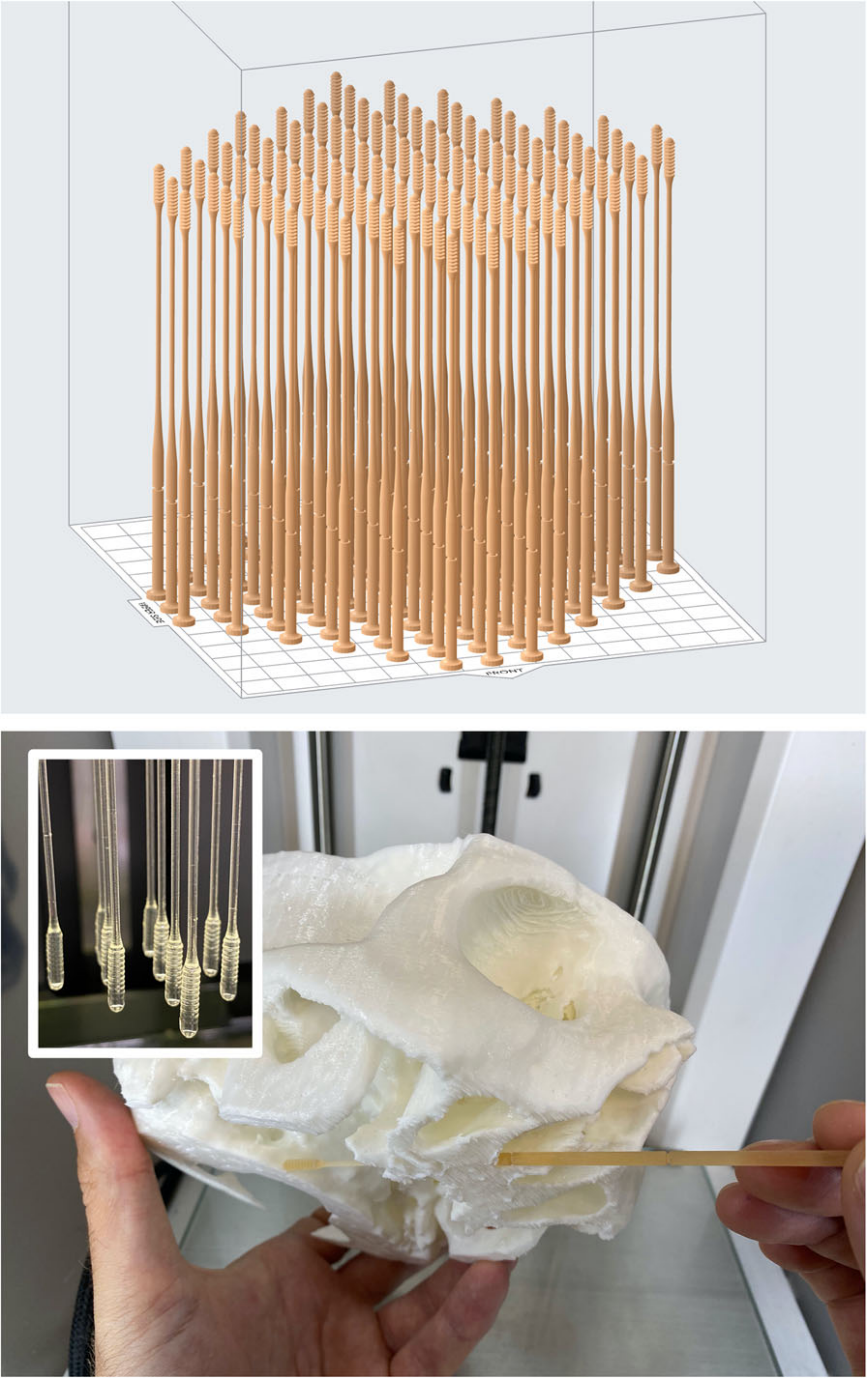
Images from the STL files and pictures of 3D printed swabs

**3. Diagnostic products and consumables** (Fig. 4). This group includes the manufacture of swabs of different designs to perform the nasopharyngeal smears necessary for the diagnosis of SARS-CoV-2 infection by PCR test. The advantage of this type of swab has been validated for clinical use [14]. The geometry of the head, the length of the handle and the position of the stigma are configurable elements that have been adapted to the different formats of the container tubes available in our Centre. Considered as a class IIa medical device and manufactured by means of stereolithography technology (SLA) with biocompatible light-curing resin, it is presented in sterile packaging in bags of 10 units and in unitary peel-packs adjusted to local needs.

Desktop 3D printing machines such as the Ultimaker S5 (Utrecht, Netherlands) or the Formlabs Form 2 (Massachusetts, USA) were used to allow decentralised, massively parallel and economic production of the devices in the pandemic environment. The following additive manufacturing technologies were used:

- FDM (Fused Deposition Modeling) in PLA plastic (24$ per kilogram). This is the most readily available technology and simple technology used for the majority of the products that don’t have further requirements.
- SLA (Stereolithography) with engineering resins (175$ per kilogram) for the parts that were required to be airtight, because of the improved resolution, density and surface finish; and Class IIa biocompatible resin (Formlabs’ BioMed Amber Resin; 300$ per kilogram) for the nasal swabs.

The products were mainly manufactured by individuals (57.3%), universities (7.6%) or associations (2.8%). Manufacturing companies have made 28% of the products, and the rest (4.3%) have been achieved through in-house manufacturing, in this last case being mostly prototypes and product validations.

The products have been donations in 78.2% of the cases. However, if a greater quantity and optimisation of the production times of a product has been required due to care needs, the acquisition has been made through public purchase. This is the case of face shields or some of the products for ventilatory support, with the purchase of 49% of the 3d printed face shields, 63% of the Charlotte adapters, 48% of the side port connectors and 8% of the Dave adapters.

The delivery date of the products started on March 25th and has been extended until the last day of the study period. Since the beginning of the period, production has been adapted to the demand made by the services in charge of both the availability, coordination and logistics of the personal protective equipment and the care needs of the clinical services involved in the ventilatory support therapies. Thus, during the first week 83.7% of the face shields had already been delivered. However, the demand for other products has been later, such as the connectors for ventilatory support delivered from 31st March, or the ear savers whose first delivery was made on 14th April.

The destination of the products delivered was the Centre itself in 90.9% of the cases, although the production capacity, together with the demand caused by the global shortage, made it possible to deliver products to other Centres (Table 1).

**Table 1:** Types of products delivered to other Centres

*Destination*	Charlotte adapter	Face Shields	Total amount
Hospital Santa Cristina (Madrid)		475	475
Clínica Ruber (Madrid)		349	349
Hospital Infanta Elena (Valdemoro)		120	120
Centro de Salud Maqueda (Madrid)		101	101
Hospital Montecelo (Vigo)		100	100
Clínica Universidad de Navarra (Madrid)		100	100
Hospital Beata María Ana (Madrid)		50	50
Hospital La Princesa (Madrid)	22		22
Hospital del Sureste (Arganda)	20		20
Hospital Alcorcón (Alcorcón)	20		20
**Total amount**	**62**	**1295**	**1357**

Regarding quality control, parts were collected from the producer network by couriers sent from the Hospital, where assembly (if needed), quality check and delivery to the users was centralised. Each delivery was checked at the arrival, parts counted and noted. In addition, a second check was performed before assembly.

## **Discussion and conclusions**

The integration of 3D printing technology in the healthcare process allows for the monitoring of the end-to-end process, from the design and validation for clinical use of different products to the optimized production of these products adapted to the healthcare need, which is of great importance in health systems during periods of sanitary crisis such as the COVID-19 pandemic, when the shortage of healthcare resources at a global level makes universal availability and coverage impossible.

The manufacturing university hospital is not born as a competition of the factories or the traditional medical industry, but it generates value in personalized medicine by gathering the professional team and the necessary resources to be able to treat by means of a personalized medical product with the maximum guarantees of quality and commitment to the patients, generating knowledge based on each individual experience, and thus allowing a qualitative leap in exponential patient-centered medicine.

Hospital General Universitario Gregorio Marañón is a pioneer in the transversal implementation of 3D printing at a hospital level, incorporating an advanced planning and POC manufacturing unit integrated as an in-house 3D printing laboratory in the care flow of more than 20 medical-surgical specialties. Our 3D printing laboratory was founded in 2015, initially manufacturing biomodels with FDM technology, and later surgical guides in resin using SLA technology. As a manufacturer, the hospital is licensed for the manufacture of medical devices and certified by the international standard ISO 13485 for Quality Management Systems for medical devices. This role as a manufacturing hospital has enabled networking and coordination in production with other hospitals and with associations, institutions or individuals with the possibility of printing products previously validated from the Centre according to care needs. In a manufacturing university hospital, 3D printing goes hand in hand with teaching and translational research, acting as an accelerator of clinical innovation and enabling advanced rapid prototyping, reducing verification and validation times. This has been the case with surgical masks or some of the parts manufactured for ventilatory support systems [15].

The appearance of different socio-cultural movements such as Coronavirusmakers [16] and collaborative networks such as the one created by the Parc Taulí Foundation [8], making available different products with the technical design specifications, material requirements and printing parameters, have made it possible to optimise the production of some of these products such as the Charlotte and Dave adapters, and other connectors and valves. Sharing this new paradigm, at the UPAM3D of our hospital we have designed and validated the clinical utility of some products such as face shields, connections that facilitate double oxygen inlet, or adaptation pieces for positive pressure ventilation systems, publishing the designs available to the community (Table 2).

**Table 2:** Name of products (rows) and file URL (columns)

**3D printed product**	**Author**	**Download link**
Face Shield	UPAM3D	https://www.thingiverse.com/thing:4258101
Ear Saver	UC3M	https://github.com/roboticslab-uc3m/covid19-fablab/blob/a6568a7eb4245d05b8781169d61f92aae42d1035/SujetaMascarillasUC3M.stl
Charlotte adapter	Hospital de Gardone Valtrompia, ISINNOVA	https://www.isinnova.it/easy-covid19-esp-2/
Hands-free door opener	Lupeon SL	https://lupeon.com/2020/03/lu-touch/
Side port connector	Hospital Parc Taulí	http://www.tauli.cat/es/institut/plataformes-i-serveis/laboratori-3d/covid-3d/cataleg/#3dpt012
Dave adapter	Hospital de Gardone Valtrompia, ISINNOVA	https://www.isinnova.it/easy-covid19-esp-2/
Dual input connector	UPAM3D	https://www.thingiverse.com/thing:4282537
CPAP adapter	UPAM3D	https://www.thingiverse.com/thing:4282700
Tube connector	UPAM3D	https://www.thingiverse.com/thing:4282640
Swabs	Hospital Virtual Valdecilla University of South Florida’s (USF) Department of Radiology	https://www.hvvaldecilla.es/proyectos/recogidas-de-muestras-hisopos/ 3dclinicalapplications@usf.edu
One-way valve	Hospital Parc Taulí	http://www.tauli.cat/es/institut/plataformes-i-serveis/laboratori-3d/covid-3d/cataleg/#3dpt023
Photophore accessory	Hospital Virtual Valdecilla	https://www.hvvaldecilla.es/proyectos/accesorio-para-fotoforos/
Mask	Cagriahiskali	https://cults3d.com/en/3d-model/tool/covid-19-mask-easy-to-print-no-support-modified-version
Venturi Nozzle	Mothy	https://www.thingiverse.com/thing:4231890
Elbow door-opener	Hospital Parc Taulí	http://www.tauli.cat/es/institut/plataformes-i-serveis/laboratori-3d/covid-3d/cataleg/#3dpt002

The disinterested offer of the different actors to participate in this type of production has made it possible both to cover the intrahospital demand and to collaborate with other centres, even at times when the need for specific products has required an increase in production.

**Fig. 5:**
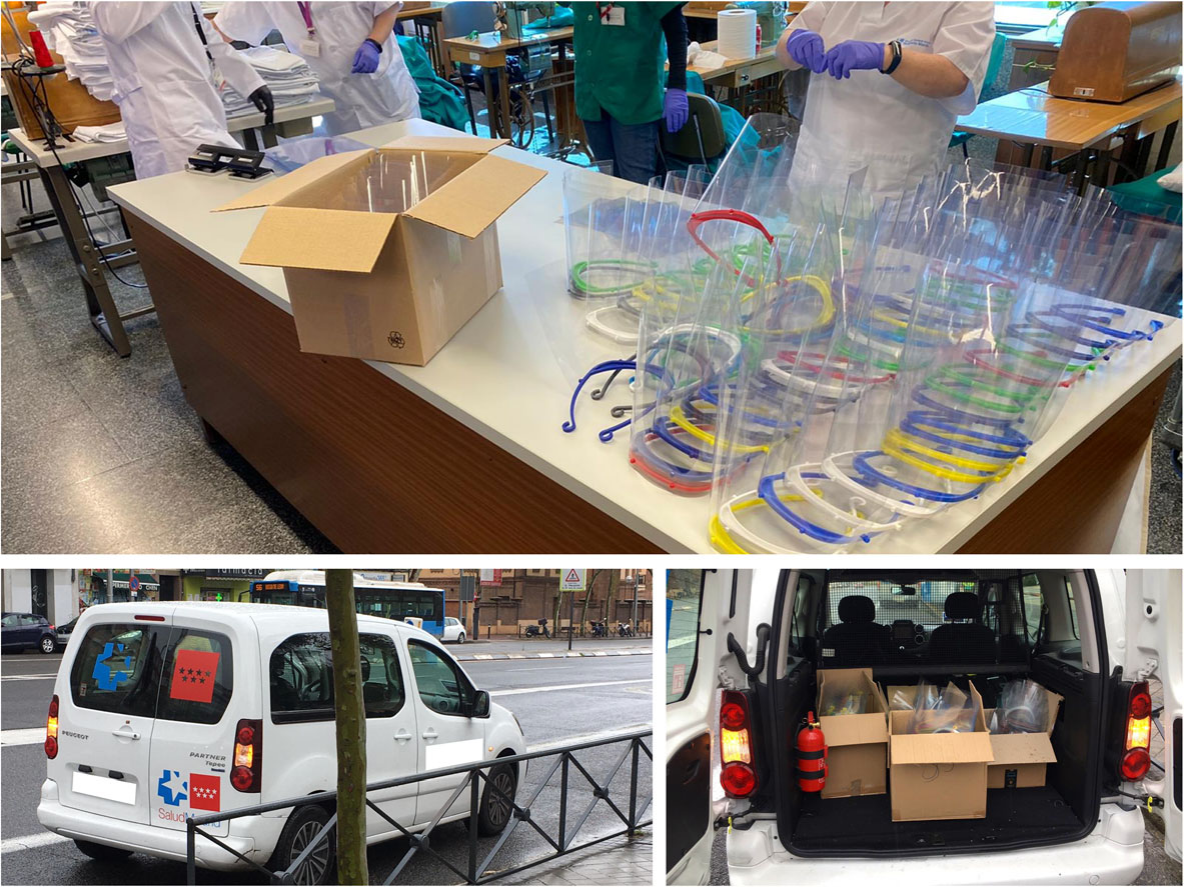
Example of distributed production. Hospital General Universitario Gregorio Marañón

It is interesting to note that the first products are delivered 11 days after the start of the alarm period. This is justifiable because in the first days of the study period, and in the face of a minor casuistry, the demand for products was also lower. In those first days, the activity as a manufacturing university hospital consisted of designing and validating prototypes as well as identifying collaborators for the constitution of a distributed production network (Fig. 5), so that incidents in terms of need and/or supply could be minimized with the most agile response possible . Thus, after 7 days, the first face shields units were manufactured and delivered under the coordination of the Services responsible for occupational risk prevention. An internal validation of the design was carried out, and after assessing the benefit-risk in the areas with more limited resources, the distribution was staggered. Something similar has occurred in the manufacture of parts for ventilatory support: when the lack and difficulty of supply from the different Clinical Services is identified, it is reported to the UPAM3D from where action is coordinated not only to respond to the high demand, but also for rapid prototyping or design improvements. The production offer of a large number of participating actors has facilitated the maintenance of the rhythm of production and collaborative work with other Centres, also allowing a staggered delivery and adjusted in real time to the demand.

During the first wave of the pandemic we had to apply emergency solutions, where the vocation and personal effort were essential. Given the exorbitant prices of products with very low stock at that time, the general feeling was one of great speed of response and access to a large number of products at a very low cost, although a cost-economic analysis is not available because it was an exceptional situation where any comparative reference price had not been extrapolated.

Although there are multiple benefits related to POC manufacturing, and even being very significant the contribution in terms of internal knowledge generation, customization, local quality control and cost reduction; we undoubtedly want to highlight the response times as the main advantage over traditional manufacturing and distribution. During this first wave of the pandemic, the pace of manufacturing and the minimum delay with the commissioning, the scalability of production and the capacity of adaptation to the exponential increase experienced, have been key factors in placing this health product manufacturing paradigm in a preferential position within the health system.

In spite of the limitation of being a descriptive case study conditioned by the activity of a third-level university hospital with a very high casuistry of patients affected by COVID-19, which has forced a restructuring of the Centre at all levels [17], the results obtained reflect that the role of the manufacturing hospital allows the reconciliation of in-house and distributed manufacturing with traditional production, providing custom-made adaptation of the specifications, as well as maximum efficiency in the working and availability of resources, which is of special importance at critical times for health systems such as the current COVID-19 pandemic.

## Data Availability

All data analyzed during the current case study are available from the corresponding author on reasonable request. The 3D files are available in the download links included in this paper.

## Abbreviations

3D: Three dimensional
COVID-19: Coronavirus disease 2019
CPAP: Continuous positive airway pressure
FDM: Fused deposition modeling
ISO: International Organization for Standardization
SARS-CoV-2: Severe acute respiratory syndrome coronavirus 2
PCR: Polymerase chain reaction
POC: point of care
PPE: Personal protective equipment
SLA: Stereolithography
